# Significance of metastatic topography for the immunotherapy of cutaneous and ocular melanomas

**Published:** 2020

**Authors:** Sorin Săftescu, Mihnea Munteanu, Dorel Popovici, Radu Dragomir, Alina Gabriela Negru, Patricia Cristina Pac, Șerban Mircea Negru

**Affiliations:** * OncoHelp Oncology Center, Timișoara, Romania; ** Department of Ophthalmology, “Victor Babeș” University of Medicine and Pharmacy, Timișoara, Romania; *** “Victor Babeș” University of Medicine and Pharmacy, Timișoara, Romania; **** Department of Cardiology, “Victor Babeș” University of Medicine and Pharmacy, Timișoara, Romania; ***** Department of Ophthalmology, County Clinical Emergency Hospital Timișoara, Romania; ****** Department of Oncology, “Victor Babeș” University of Medicine and Pharmacy, Timișoara, Romania

**Keywords:** melanoma, ocular, nivolumab, metastases

## Abstract

PD-1 is expressed on the surface of activated T lymphocytes and belongs to the category of negative immune stimuli. Its blocking stimulates the immune response to tumor antigens. Ocular melanomas represent 3-4% of the total melanomas and have metastatic potential, especially to the liver but also to the lungs, skin, and bones. In the case of metastatic melanoma, immunotherapy has a unique role due to the lower frequencies of BRAF mutation in choroidal melanomas and consecutive exclusion from treatment with specific BRAF tyrosine kinase inhibitors. A retrospective observational study was performed in 42 patients who received immunotherapy (IT) with nivolumab for cutaneous and ocular metastatic melanomas, aimed at highlighting the association between the topography of metastases and the duration of immunotherapy until progressive disease. The results indicated the presence of liver metastases as a negative predictive factor for IT duration in patients with melanoma and the presence of lymphatic metastases as a predictive factor for longer IT in patients with melanoma under 65 years old.

## Introduction

The efficient functioning of the immune system requires PD-1 (“programmed death – 1”), which is a surface receptor expressed on activated T lymphocytes. PD-1 belongs to the category of negative immune stimuli. Nivolumab binds with high affinity to the PD-1 receptor and prevents interaction with PDL-1 and PDL-2 ligands, stimulating the memory immune response to tumor antigens and the proliferation of antigen-specific T cells [**1**,**2**].

The mean half-life of nivolumab was estimated at 25 days, which means at least 12 weeks of treatment until a serum plateau concentration is reached [**3**]. Other estimates provide half-lives for nivolumab of 12-20 days [**4**].

Degradation of anti-PD-1 antibodies appears to be achieved by mechanisms of proteolytic degradation belonging to liver cells or reticuloendothelial system. Degradation rates are unaffected by age, tumor size, renal dysfunction, or mild liver dysfunction. The higher rate of degradation of these antibodies correlates with a weaker response to treatment. The low level of albuminemia at the start of treatment is correlated with increased clearance of nivolumab and a weaker therapeutic response [**5**-**7**].

Administration of a single dose of 0.3 mg/kg of nivolumab results in a PD-1 receptor occupancy level of 65% while a dose of 3.0 mg/kg results in an occupancy level of only 69% and a dose of 10 mg/kg induced an occupancy level of 70% (estimates valid for circulating cells) [**8**].

The effects of neutralizing antibodies under treatment remain to be evaluated. Anti-nivolumab antibodies occur in 11-37.8% of the treated cases - especially in combination with ipilimumab - and neutralizing antibodies have been documented in up to 4.6% of the cases [**9**].

Risk factors for the development of ocular melanomas are: Caucasian race, light eyes (blue, green), advanced age, the association of cutaneous dysplastic nevus syndrome, oculodermal melanocytosis, presence of cutaneous or ocular nevus, exposure to natural or excessive artificial light [**10**].

Ocular melanomas represent 3-4% of all melanomas. Topographically, ocular melanoma may have as a starting point the choroid (most commonly) and uvea (especially iris). The incidence of the disease is 5 cases per year per 1 million population, and the cell of origin is the melanocyte. Ocular melanoma has metastatic potential, especially to the liver but also to the lungs, skin and bones. Sometimes, metastases are detected years after the primary disease.

The symptoms of ocular melanomas are not always present and may include: photopsia, unilateral blurry vision, diplopia, pupil irregularities, loss of peripheral vision, a growing dark spot on the iris, pain, foreign body sensation, metamorphopsia (distortion of vision) [**11**].

The rare frequency of BRAF driver mutation in choroidal melanomasis particular for ocular melanoma. The situation of iridal melanoma is different because it proves a prevalence of BRAF mutation of about 50%, comparable to the frequency of the same mutation among cutaneous melanomas of 60-65% [**12**].

## Materials and methods

A retrospective observational study included patients receiving immunotherapy (IT) with nivolumab for cutaneous and ocular metastatic melanomas, which aimed to highlight the associations between the topography of metastases and the duration of immunotherapy.

Thus, 2241 hospitalizations (especially day-care) were processed for the administration of immunotherapy belonging to 220 distinct persons between 28th of June 2017 and 20th of March 2020. Among the 220 cases, 42 cases of melanoma were identified (with 527 IT administrations) of which 2 cases of ocular melanoma (4.7% of the treated metastatic melanomas).

Since the distribution of immunotherapy durations is not normal (Gaussian), the Mann-Whitney U Test (MWUT) was applied for sets of individual variables and the correlations between IT duration and various parameters were verified with Cox Proportional Hazards Survival Regression (CPHSR) which takes into account both cases still in treatment as well as those out of treatment.

## Results

For the entire group of 220 patients, the mean duration of immunotherapy (discontinued due to lack of clinical benefit/disease progression/intolerance) is 163.60 days. Remarkably, 95 cases (of which 18 with melanoma) are still under treatment (they have been administered over the last 3 weeks compared to the cut-off date) (**Table 1**). Single-dose patients were counted with 0 duration of treatment. The average duration of treatment is 194.55 days if only cases with at least two administrations are considered. The maximum duration recorded is 783 days (patient with melanoma, treatment still in progress).

**Table 1:** Distribution of cases by cancer site

Type of cancer	Number of cases	Percentage	Average age	Number of cases still in treatment	Percentage
lung	154	70,0	61,97	66	69,5
melanoma	42	19,1	60,90	18	18,9
renal	24	10,9	59,17	11	11,6
Total cases	220			95	

**Fig. 1:**
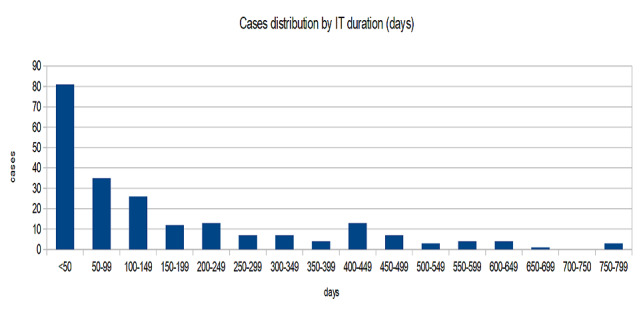
Distribution of cases by the duration of immunotherapy

The distribution of the duration of immunotherapy is a particular one, the curve of the number of cases that have gone through a specific duration of immunotherapy has a hyperbolic aspect with a secondary peak in the area of 400-449 days (**Table 2**). Out of the 220 cases, 35 underwent only one administration, and the other 21 did not exceed one month of treatment (**Fig. 1**). The median duration of treatment was 89 days.

**Table 2:** Distribution of cases by the duration of immunotherapy

IT duration (days)	<50	50-99	100-149	150-199	200-249	250-299	300-349	350-399
number of cases	81	35	26	12	13	7	7	4
IT duration (days)	400-449	450-499	500-549	550-599	600-649	650-699	700-750	750-799
number of cases	13	7	3	4	4	1	0	3

The mean duration of immunotherapy was longer for melanomas and renal cancer compared to lung cancer (considering only cases initiated after reimbursement of treatment for all locations) (**Table 3**). MWUT revealed a value of p = 0.05155 extremely close to the statistical validation of the significant difference in the individual durations of immunotherapy in melanomas and lung cancers.

**Table 3:** The average duration of immunotherapy according to the cancer site

Initiated cases for all locations	Average IT duration (days)
lung cancer	146,13
melanoma	209,75
renal	202,57

The 42 cases of melanoma were divided into 26 males and 16 females (61.9% and 38.1% of the cases, respectively). The number of IT initiations was 6 for the period of 6 months in 2017, 16 cases during 2018, 17 cases in 2019 and 3 cases in the first two and a half months of 2020, which indicated an average of 1.27 patients initiated monthly with IT for melanoma in our center. The treated cases of ocular melanoma belonged to two females, both aged 62 years at initiation, treated for 63 and 97 days, respectively, until progressive disease.

**Fig. 2:**
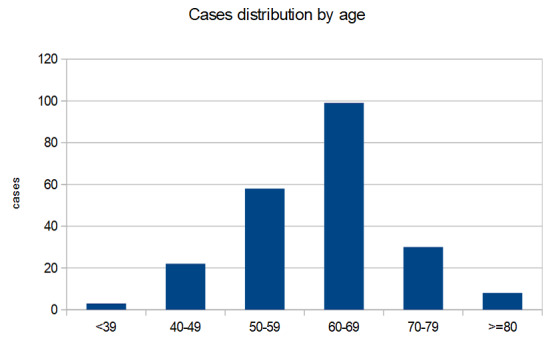
Distribution of cases by age groups

The data for the whole 220 patients group under IT revealed data similar to those presented above: the distribution by sex revealed 155 cases (70.5%) belonging to male gender and 65 cases (29.5%) belonging to female gender. The average duration of immunotherapy was 155.12 days for males and 183.83 days for females. The CPHSR statistical test could not validate as significant the difference between the genders of the IT durations (p = 0.936). The average age of the group of patients enrolled was 61.45 years, with small differences in groups with different locations of neoplasms (**Fig. 2**).

The age distribution of melanoma cases (42) revealed the dominance of the seventh decade (60-69 years). Of note were two cases aged 18 and 33 years, respectively, and 4 cases initiated at the age of over 80 years (9.5% of the group) (**Table 4**).

**Table 4:** Distribution of melanoma cases by age groups

Distribution by age	Number of cases	Percentage
<39	3	7,1
40-49	7	16,7
50-59	6	14,3
60-69	14	33,3
70-79	8	19
>=80	4	9,5

**Fig. 3:**
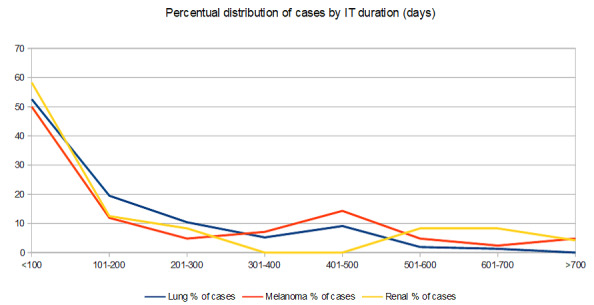
Distribution of cases according to location and duration of immunotherapy

Fractions of cases exceeding 300 days of treatment were higher for kidney cancer and melanomas (**Fig. 3**).

The frequency of use of mild and strong opioids (WHO category II and III) was 4.8% among melanoma cases, 8.4% among lung cancer cases and 8.3% among renal cancer cases, respectively. Opioid use was not associated with significantly reduced average treatment times (**Table 5**).

**Table 5:** Average duration of immunotherapy according to opioid use

Average IT duration by opioid use	Average IT duration	Cases
no opioids	163,9	203
WHO category II or III	160,4	17

**Fig. 4:**
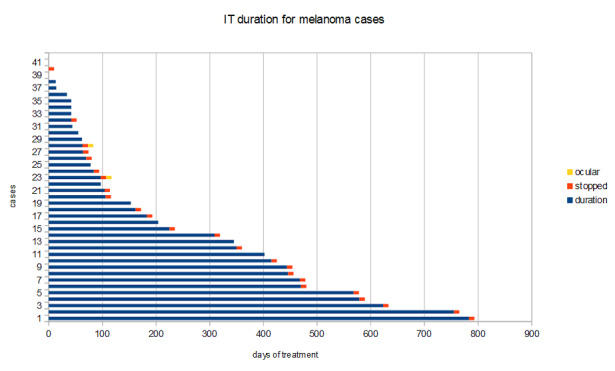
Cases of nivolumab-treated melanoma stratified by IT duration

An overview of the duration of IT, marking the cessation of treatment (**Fig. 4**):

Cox Proportional Hazards Survival Regression was applied to different groups of cases, investigating the presence of liver and bone metastases. Lymphatic and adrenal glands as shorter IT risk factors in patients with melanoma (negative coefficients translate into shorter IT durations). **Table 6** includes the determinants for IT duration identified with values of p <0.1. Interpretation for the relative risk is coefficient; if the coefficients are negative: the duration of IT increases with the determinant (favorable predictive factors); positive coefficients: IT duration decreases as the determinant increases. Liver metastases were identified in 40.4% of treated melanoma patients, bone metastases in 11.2%, lymphatic patients in 57.1% and adrenal metastases in 9.5%.

**Table 6:** Coefficients of variation for IT duration and statistical significance by different patient groups

Patient group	Parameter	Number of cases	Coefficient	Statistical significance	Confidence interval	Risk Ratio	Confidence interval
Melanoma all cases	Liver metastases present 0/1	42	1.36	P=0.0086	0.34 to 2.38	3.91	1.41 to 10.85
Melanoma, cases > 65 years	Age (years)	18	-0.13	P=0.0773	-0.28 to 0.01	0.87	0.75 to 1.01
Melanoma, cases ≤ 65 years	Lymph node metastases present 0/1	22	-1.99	P=0.0330	-3.82 to 0.16	0.13	0.02 to 0.85

## Discussions

The half-life of nivolumab of 25 days and the time required to reach the serum concentration plateau of 84 days raised the question of the usefulness of a loading dose, considering that 43% of the patients with melanoma have a treatment duration with nivolumab shorter than 84 days.

The number of cases in the analyzed subsets proved to be relatively small to allow statistical conclusions for most iterations, leading to uncomfortably wide confidence intervals.

Melanoma cases represented 19.1% of all immune treated cases. Among them, the cases of ocular melanoma, however, painted a more aggressive profile for the evolution of the disease: an average IT duration of 80 days for ocular melanoma cases compared to the average of 215 days of IT duration among the 42 cases of melanoma (even though we had extremely few cases of ocular melanoma).

Surprisingly, among the total of 220 patients, the cases following opioid treatment (WHO category II or III) revealed only slightly different averages of IT duration, the number of cases not allowing statistical conclusions to the Mann Whitney analysis or Cox regression (to be mentioned however, CPHSR revealed a coefficient of 0.4053 with a relative risk of exit from IT of 1.49 [0.85 to 2.61] for those under opioid treatment, but with a p value of 0.1539).

## Conclusions

The presence of liver metastases is a negative predictive factor for the duration of IT in patients with melanoma (p <0.05). Lymphatic metastases are a predictive factor for longer IT in melanoma patients under 65 years old (p <0.05). It is necessary to extend the study in order to be able to validate the determinants currently close to the limit of statistical significance.
